# Meta-analysis of the relationship between montelukast use and neuropsychiatric events in patients with allergic airway disease

**DOI:** 10.1016/j.heliyon.2023.e21842

**Published:** 2023-11-08

**Authors:** Yakui Mou, Qing Song, Chunying Zhao, Han Fang, Chao Ren, Xicheng Song

**Affiliations:** a Department of Otorhinolaryngology Head and Neck Surgery, Yantai Yuhuangding Hospital, Qingdao University, Yantai, Shandong Province, 264000, China; b Shandong Provincial Clinical Research Center for Otorhinolaryngologic Diseases, Yantai, Shandong Province, 264000, China; c Yantai Key Laboratory of Otorhinolaryngologic Diseases, Yantai, Shandong Province, 264000, China; d Department of Otolaryngology, Yantai YEDA Hospital, Yantai, Shandong Province, 264000, China; e Epilepsy Sleep Center, Linyi People Hospital, Linyi, Shandong Province, 276000, China; f Department of Neurology, Yantai Yuhuangding Hospital, Qingdao University, Yantai, Shandong Province, 264000, China

**Keywords:** Allergic airway disease, Montelukast, Neuropsychiatric event, Children, Allergic rhinitis, Asthma

## Abstract

Use of montelukast, as a cause of neuropsychiatric events, in patients with asthma or allergic rhinitis is controversial, and comprehensive statistical analyses verifying this relationship remain lacking. To better understand the relationship between montelukast and neuropsychiatric events, it is vital to guide patients in the effective use of the drug, especially in children whose mothers are concerned about its side effects. In this study, randomized controlled trials (RCTs) investigating montelukast and neuropsychiatric events were retrieved from a literature search of the Medline (1966 to February 2023), Embase (1974 to February 2023), Web of Science, and other related databases. After screening, 18 RCTs were ultimately included in a meta-analysis to merge statistics, which demonstrated no significant increase in neuropsychiatric events compared with placebo. A similar pattern of adverse neuropsychiatric events was observed in patients grouped according to age, with headache being the most common adverse neuropsychiatric event. Overall, montelukast did not significantly increase neuropsychiatric events in patients with allergic rhinitis and/or asthma compared with placebo. Large-sample RCTs are needed to verify the association between neuropsychiatric events and montelukast use in children, and attention is also devoted to FDA warnings.

## 1 Introduction

As a widely used leukotriene receptor antagonist, montelukast can alleviate the symptoms of allergic diseases, including asthma and allergic rhinitis (AR), and improve patient prognosis by reducing the production of inflammatory mediators [[1], [2], [3]]. Moreover, montelukast can be administered to both adults and children. However, with its widespread use, neuropsychiatric events (NEs) in patients receiving montelukast have significantly increased [[4], [5], [6], [7], [8], [9], [10], [11], [12], [13], [14]].

Considering pharmacovigilance studies [[4], [5], [6],^8^,[11], [12], [13], [14]] and case reports [7,9,10] addressing neuropsychiatric adverse reactions, the United States Food and Drug Administration (FDA) has added a warning about NEs to the montelukast label. Before prescribing montelukast, patient condition should be comprehensively assessed, including whether the patient has depression, headache, anxiety, insomnia, aggression, suicidal ideation, or behavioral neuropsychiatric adverse reactions [15,16]. In 2020, the FDA issued the following black box warning: “limit the use of allergic rhinitis unless the patient is irresponsive to or unable to tolerate other clinical drugs and comprehensively consider asthma patients before using montelukast” [17]. This has raised serious concerns about NEs among mothers, especially those whose children have allergic diseases, such as asthma and/or AR.

The relationship between NEs and montelukast currently remains controversial. Some studies have reported that patients using montelukast experience a high incidence of NEs, whereas others have concluded that the occurrence of NEs is not related to montelukast [[4], [5], [6], [7], [8], [9], [10], [11], [12], [13], [14]]. In addition, statistical analyses of the relationship between montelukast and NEs remain lacking [18]. As such, this study aimed to clarify the relationship between montelukast and the occurrence of NEs in adults and children with asthma and AR through a meta-analysis of high-quality randomized controlled trials (RCTs).

## 2 Materials and methods

Registration for the current project was completed on the INPLASY platform after the search (INPLASY202310051).

### 2.1 Search strategy

To analyze the possibility of NEs, a comprehensive survey of patients with AR or asthma who received montelukast was performed. A literature search of the Medline (1966 to February 2023), Embase (1974 to February 2023), and Web of Science databases for studies investigating montelukast and neuropsychiatric events was performed. Key words used in the literature search included: ((((((((montelukast) OR (MK 0476)) OR (MK-0476)) OR (Singulair)) OR (montelukast sodium)) OR (sodium 1-(((1-(3-(2-(7-chloro-2-quinolinyl)ethenyl)phenyl)-3-(2-(1-hydroxy-1-methylethyl)phenyl)propyl)thio)methyl)cyclopropylacetate))) OR (leukotriene receptor antagonists) AND ((((((((asthma) OR (Asthmas)) OR (Bronchial Asthma)) OR (Asthma, Bronchial)) OR (Rhinitis, Allergic)) OR (Allergic Rhinitides)) OR (Rhinitides, Allergic)) OR (Allergic Rhinitis))) AND ((((((randomized controlled trial) OR (randomized)) OR (placebo)) OR (random and allocation)) OR (random allocation)) OR (randomized and controlled and trial)). In addition, the reference lists of the retrieved studies were manually searched for additional, potentially eligible, relevant studies to ensure comprehensive inclusion.

### 2.2 Inclusion and exclusion criteria

Studies were included according to the “PICOS” criteria, as follows: population of interest (P), patients with AR and/or asthma; intervention (I), patients using montelukast; comparison (C), placebo group(s) included as comparators; outcomes of interest (O), adverse drug reactions (ADRs) of montelukast; and study design (S), RCTs.

Studies involving patients with other diseases, those for which full text versions were not available online, those not published in English, and those that did not report available data for analysis were excluded.

**Fig. 1:**
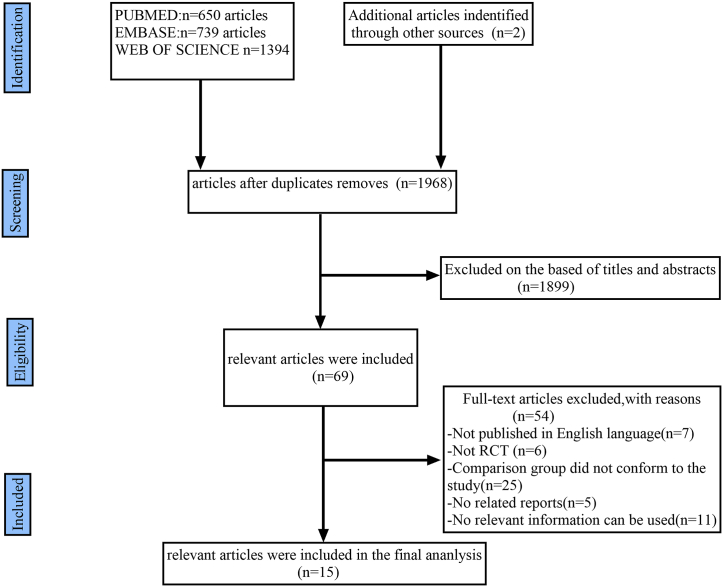
PRISMA flow diagram demonstrating the progress of study evaluation throughout the review.

A flow-diagram illustrating the study inclusion process is presented in Fig. 1.

### 2.3 Quality assessment

The quality of the included studies was assessed using the Jadad Scale [19]. Each study was assessed by certain methods (method of patient random allocation, concealment of allocation, blinding, and data loss to follow). The Cochrane Handbook for Systematic Reviews of Interventions version 5.1.0 [20] was used to grade individual studies. Each study was assigned a category, as follows: A (low risk of bias, study met almost all criteria); B (moderate risk of bias, study met part or was unclear for ≥1 quality criteria); and C (high risk of bias, study did not meet or included the criteria). Differences were resolved through discussion among the authors.

### 2.4 Data extraction

Measurable data were independently extracted and summarized by two researchers. The following data were extracted from the included studies: first author's name; publication year; names of diseases and type(s) of design; sample size(s) and patient age; therapy patients in the experimental group received; number and type of NE(s) occurrences; and follow-up period.

### 2.5 Outcome measures

The outcome measures are described in Appendix A.

### 2.6 Statistical analysis

Statistical analysis was performed using STATA version 12.0 (StataCorp LLC, College Station, TX, USA). Fixed- or random-effects models were used to combine statistics. The relative risk (RR) with corresponding 95 % confidence interval (CI) was used to estimate dichotomous outcomes. The I^2^ test and χ^2^-based Q statistics were used to assess study heterogeneity. Studies with an I^2^ value > 50 % or p < 0.1, were considered to have significant heterogeneity, and the fixed-effects model was used; otherwise, a random effects model was used. Differences and associations with P < 0.05 were considered to be statistically significant.

## 3 Results

### 3.1 Characteristics of the included studies and participants

The initial literature search retrieved 2785 studies from the databases and reference lists of retrieved studies. After screening, 15 studies were ultimately included [1,[21], [22], [23], [24], [25], [26], [27], [28], [29], [30], [31], [32], [33], [34]] containing 18 RCTs for the meta-analysis after reading the full text. The screening process and characteristics of each study are reported in Fig. 1 and Table 1, respectively.

**Table 1:** Characteristics of the included studies.

Study	Design	Disease	Age (Range, years)	Therapy in experimental group	Therapy in control group	Dosage	Sample size		NE^a^ sample size		NE^a^ type and sample size	Follow up period (wk)
							Experimental	Control	Experimental	Control		
Leff 1998	RCT^b^	asthma	15–45 years	Montelukast	Placebo	10 mg	54	56	11	18	Headache11/18	12
Knorr 1998	RCT^b^	asthma	6- 14-years	Montelukast	Placebo	5 mg	201	135	38	29	Headache38/29	8
Noonan 1998	RCT^b^	asthma	18–65 years	Montelukast	Placebo	2mg/10mg/50 mg	212	69	18	10	Headache18/10	3
Reiss 1998	RCT^b^	asthma	≥15 years	Montelukast	Placebo	10 mg	408	273	75	61	Headache73/57; depression1/4; anxiety1/0	12
Malmstrom 1999	RCT^b^	asthma	≥15 years	Montelukast	Placebo	10 mg	387	257	68	40	Headache68/40	12
Meltzer 2000–1	RCT^b^	Allergic rhinitis	15–75 years	Montelukast	Placebo	10 mg	185	91	12	6	Headache12/6	2
Meltzer 2000–2	RCT^b^	Allergic rhinitis	15–75 years	Montelukast/Loratadine	Loratadine	10 mg + 10 mg:10 mg	90	92	1	8	Headache1/8	2
Nayak 2002–1	RCT^b^	Allergic rhinitis	15–85 years	Montelukast	placebo	10 mg	155	149	5	7	Headache5/7	2
Nayak 2002–2	RCT^b^	Allergic rhinitis	15–85 years	Montelukast/Loratadine	Loratadine	10 mg + 10 mg:10 mg	302	301	9	12	Headache9/12	2
Baumgartner 2003	RCT^b^	asthma	≥15 years	Montelukast	Placebo	10 mg	313	103	31	18	Headache31/18	6
Baena-Cagnani 2003	RCT^b^	Allergic rhinitis and asthma	≥15 years	Montelukast	Placebo	10 mg	311	302	11	11	Headache11/11	4
Yildirim 2004	RCT^b^	asthma	mean:36.93 ± 2.98 years	Montelukast/Budesonide	Budesonide	10 mg + 400 mg:800 mg	15	15	2	0	Headache2/0	6
Asthma linical Research enters 2007	RCT^b^	asthma	≥15 years	Montelukast	Placebo	10 mg	164	164	34	36	nervousness34/36	24
Shah 2006	RCT^b^	asthma	18–60 years	Montelukast/Budesonide	Budesonide	10 mg + 200 mg:400 mg	30	30	1	0	Headache1/0	8
Bisgaard 2009	RCT^b^	Allergic rhinitis	2–14 years	Montelukast	Placebo	5 mg	280	133	9	2	Headache9/2	8
Chen 2021	RCT^b^	Allergic rhinitis	18–60 years	Montelukast/Budesonide	Budesonide	10 mg + 256ug:256ug	23	23	0	0	No	2
Prenner 2010–1	RCT^b^	asthma	15–92 years	Montelukast	Placebo	10 mg or 20 mg	555	1111	6	19	Headache6/19	2
Prenner 2010–2	RCT^b^	asthma	15–92 years	Montelukast/Loratadine	Loratadine	10 mg + 10 mg:10 mg	969	971	66	85	Nervous system disorders66/85; Headache34/29; Somnolence25/10	2

a NE: Neuropsychiatric event.

b RCT: randomized controlled trial.

### 3.2 Quality of individual studies

All studies were RCTs; the quality of individual studies is summarized in Table 2. The study by Prenner [32] had a Jadad level C, which indicated a high risk and may have introduced bias in the results if included; as such, it was excluded in the analysis. The quality level of four studies [22,28,31,34] were rated B, and the remaining studies [1,21,[23], [24], [25], [26], [27],^29^,^30^,^33^] were rated A (11 RCTs [1,[21], [22], [23], [24], [25], [26], [27],^30^,^31^,^33^] that enrolled patients in montelukast or placebo groups; and five RCTs [25,26,28,29,34] that enrolled patients in experimental and control groups). These RCT trials were included.

**Table 2:** Quality assessment of individual study.

Study	Allocation sequence generation	Allocation concealment	Blinding	Lost to follow-up	Calculation of sample size	Statistical analysis	Level of quality	ITT‡ analysis
Leff 1998	B	A	A	1	Yes	ANOVA † Fisher's exact test	B	Yes
Knorr 1998	A	A	A	2	Yes	ANOVA † Shapiro-Wilks test Cochran-Mantel-Haenszel test	A	Yes
Noonan 1998	A	A	A	0	Yes	ANOVA † Fisher's exact Cochran-Mantel-Haenszel test Tukey's modified linear trend test (stepwise trend test)	A	Yes
Reiss 1998	A	A	A	11	Yes	ANOVA † Cochran-Mantel-Haenszel test	A	Yes
Malmstrom 1999	A	A	A	13	Yes	ANOVA † Dunnet–Tamhane approach, Shapiro–Wilk statistic, Levene test	A	No
Meltzer 2000-1	A	A	A	4	Yes	ANOVA † Fisher exact test Cochran-Mantel-Haenszel test;	A	No
Meltzer 2000-2	A	A	A	4	Yes	ANOVA † Fisher exact test Cochran-Mantel-Haenszel test;	A	No
Nayak 2002-1	A	A	A	3	Yes	ANOVA † Wilcoxon rank sum test	A	Yes
Nayak 2002-2	A	A	A	3	Yes	ANOVA † Wilcoxon rank sum test	A	Yes
Baumgartner 2003	A	A	A	NA	Yes	ANOVA †	A	Yes
Baena-Cagnani 2003	A	A	A	0	Yes	ANOVA †	A	Yes
Yildirim 2004	A	A	B	0	Yes	Mann–Whitney *U* test	B	No
Asthma Clinical Research Centers 2007	A	A	A	NA	Yes	Huber-White variance; Linear and logistic regression models	A	Yes
Shah 2006	A	A	A	0	Yes	ANOVA †	A	Yes
Bisgaard 2009	B	B	A	0	Yes	Fisher's exact test	B	No
Chen 2021	A	B	B	5	Yes	χ^2^ tests Pearson correlation test T test	B	No
Prenner 2010-1	B	B	C	9	Yes	NA§	C	No
Prenner 2010-2	B	B	C	13	Yes	NA§	C	No

A, low risk of bias: the study met almost criteria; B, moderate risk of bias: the study met part or unclear for one or more quality criteria; C, high risk of bias: the study was not met or included criteria; †ANOVA: analysis of variance; ‡ITT: Intention-to-treat; §NA: not available.

### 3.3 NEs

#### 3.3.1 The occurrence of NEs in randomized placebo-controlled studies

**Fig. 2:**
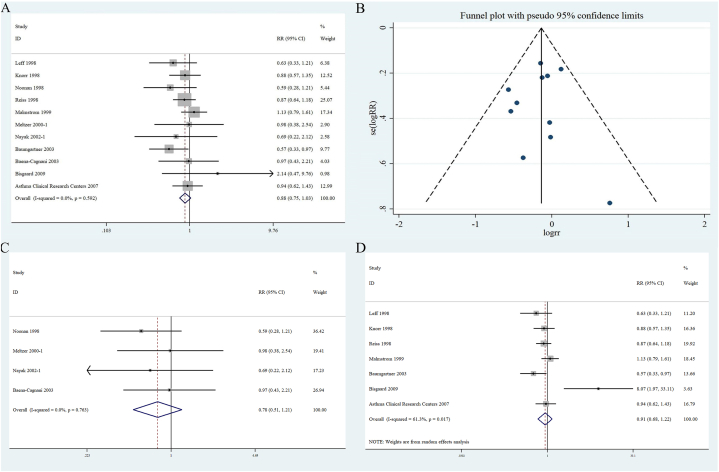
Forest plots of (A)：the relationship between patient with asthma or allergic rhinitis use montelukast and the occurrence of neuropsychiatric events; (B): funnel plot of the included studies; (C)：the relationship between montelukast and neuropsychiatric events in <1 month group; (D)：the relationship between montelukast and neuropsychiatric events in≤ 1 month group.

Eleven RCTs [1,[21], [22], [23], [24], [25], [26], [27],^30^,^31^,^33^] that enrolled 4402 patients (montelukast, n = 2670; placebo, n = 1732) were used to assess the occurrence of NEs in patients with AR and/or asthma. No significant heterogeneity (I^2^ < 50 %, P > 0.1) was found. Therefore, a fixed-effects model was used to consolidate the statistics and RR was used to assess the effect size (Fig. 2A). The forest plot revealed no statistical significance (RR 0.88 [95 % CI 0.75–1.03]; P = 0.592) in the difference between NEs in the experimental and control groups. A funnel plot developed to assess the publication bias of NEs in patients receiving montelukast or placebo presented a symmetrical appearance suggesting low publication bias (i.e., not statistically significant [Begg test, P = 1.00; Egger's test, P = 0.70]) (Fig. 2B).

To further evaluate the effect of follow-up on NE occurrence, patients were further divided into two groups (<1 month and ≥1 month). The <1 month group included 4 RCTs [1,23,25,26] with 1474 patients (montelukast, n = 863; placebo, n = 611). The forest plot revealed no statistically significant association between the NEs and montelukast (RR 0.78 [95 % CI 0.51–1.21]; P = 0.763). The ≥1 month group included 7 RCTs [21,22,24,27,30,31,33] with 2928 patients (montelukast, n = 1807; placebo, n = 1121). The forest plot demonstrated a statistically significant association between NEs and montelukast (RR 0.91 [95 % CI 0.68–1.22]; P = 0.017) (Fig. 2C and D).

#### 3.3.2 Headache-related NEs

**Fig. 3:**
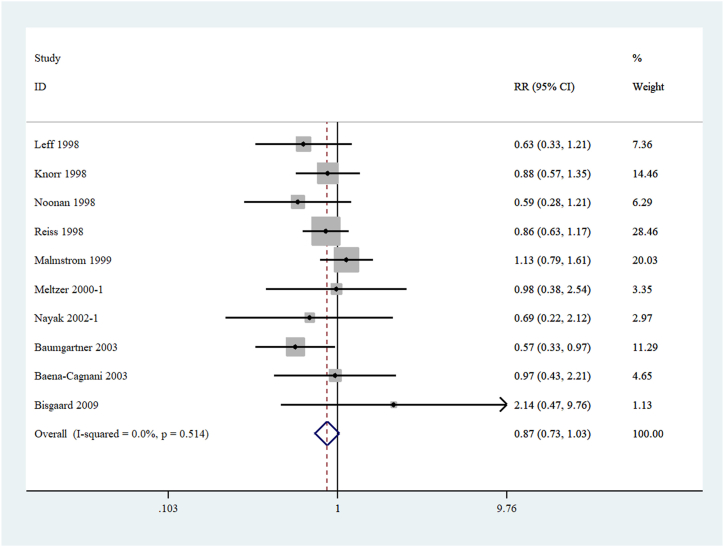
Forest plots of patients with asthma or rhinitis have been linked to a neuropsychiatric event called headache.

Ten RCTs [1,[21], [22], [23], [24], [25], [26], [27],^31^,^33^] that enrolled 4074 patients (montelukast, n = 2506; placebo, n = 1568) were used to assess the occurrence of headache, the most common event in NEs. No significant heterogeneity was found; thus, a fixed-effects model was used. The results indicated no statistically significant difference in the occurrence of headaches between the montelukast and placebo groups (RR 0.87 [95 % CI 0.73–1.03]; P = 0.514) (Fig. 3).

#### 3.3.3 NEs in patients with asthma or AR

**Fig. 4:**
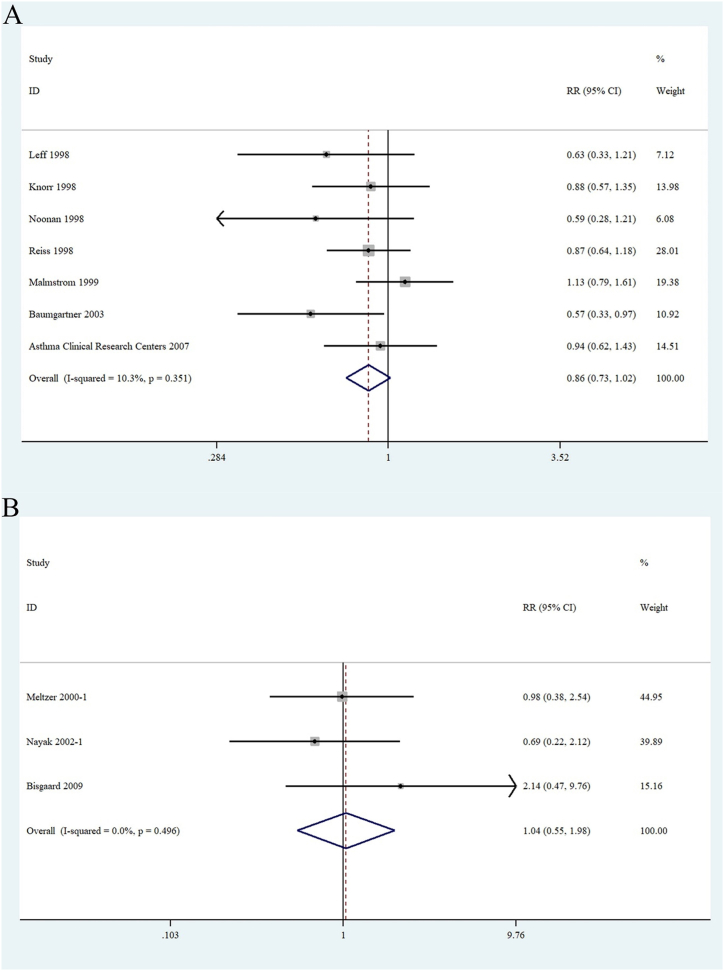
Forest plots of (A): the relationship between the use of montelukast and neuropsychiatric events in asthma patients and (B) the relationship between the use of montelukast and neuropsychiatric events in patients with rhinitis.

Asthma and AR are common chronic inflammatory diseases. Seven RCTs [[21], [22], [23], [24],^27^,^30^,^33^] that enrolled 2796 patients (montelukast, n = 1739; placebo, n = 1057) reported data regarding the occurrence of NEs in patients with asthma. The study found no statistically significant differences between the montelukast and placebo group (RR 0.86 [95 % CI 0.73–1.02]; P = 0.35) (Fig. 4A). Three RCTs [25,26,31] evaluated ADRs to montelukast in 993 patients with AR (montelukast, n = 620; placebo, n = 373). Results of analysis suggested that there was no increase in NEs in patients with AR who received montelukast compared with the placebo group (RR 1.04 [95 % CI 0.55–1.98]; P = 0.496) (Fig. 4B).

#### 3.3.4 NE in children or adults

**Fig. 5:**
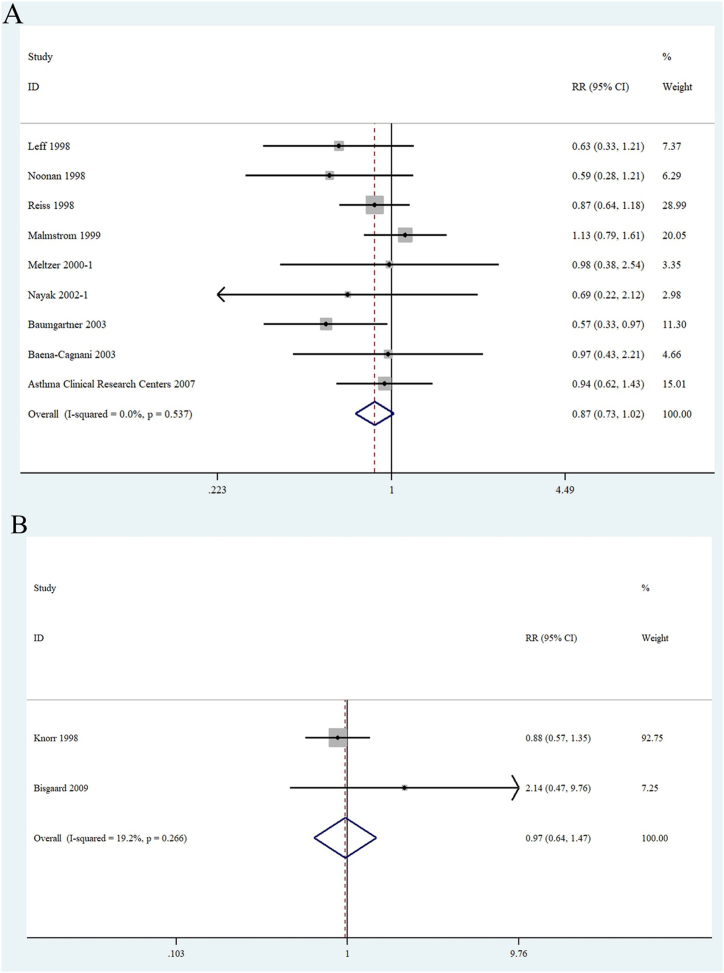
Forest plots of (A)：the relationship between montelukast and neuropsychiatric events in adult patients with asthma or rhinitis and (B): the relationship between montelukast and neuropsychiatric events in child's patients with asthma or rhinitis.

Nine RCTs [1,[22], [23], [24], [25], [26], [27],^30^,^33^] that enrolled adult 3653 patients (≥15 years of age) (montelukast, n = 2189; placebo, n = 1464) were used to estimate the occurrence of NEs. Forest plots for the fixed-effect model indicated an RR of 0.87 (95 % CI 0.73–1.02; P = 0.537) (Fig. 5A), with no statical associations between the two groups. Two RCTs [21,31] with 749 child patients (≤14 years of age) evaluated the occurrence of NEs (montelukast, n = 481; placebo, n = 268). Based on results from the fixed-effect model, the result revealed no increase of the number of NEs in child patients taking montelukast compared with placebo (RR 0.97 [95 % CI 0.64–1.47]; P = 0.266) (Fig. 5B).

#### 3.3.5 Relationship between NE and montelukast or common clinical drugs

**Fig. 6:**
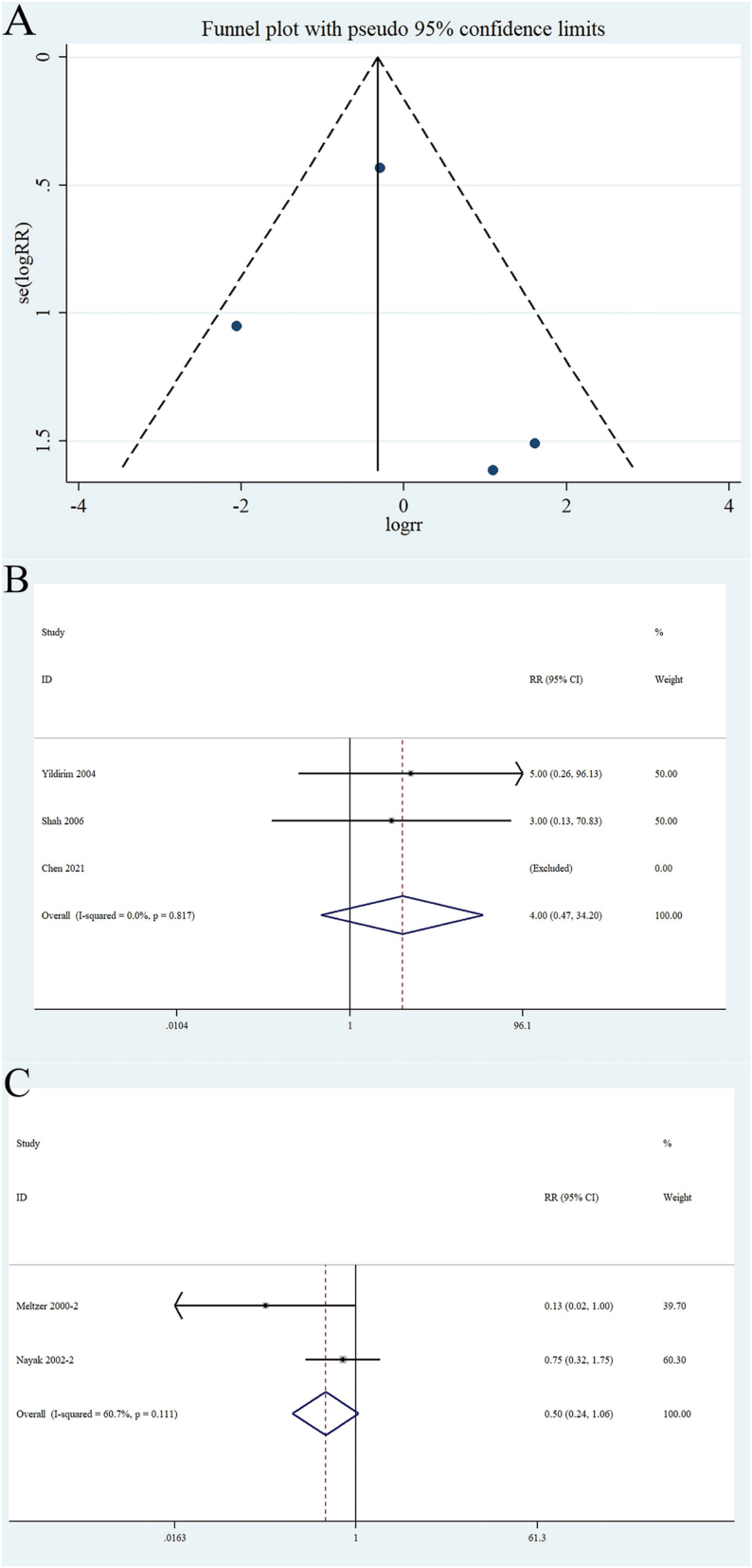
Funnel plot of (A): the included studies based on the patient with montelukast or common clinical drugs analysis and forest plots of (B)：the neuropsychiatric events between montelukast and budesonide versus budesonide alone in asthmatic patients and (C): the occurrence of neuropsychiatric events in patients with allergic rhinitis compared with montelukast combined with loratadine versus loratadine alone.

Five RCTs [25,26,28,29,34] enrolling 916 patients (experimental, n = 458; control, n = 458) assessed the occurrence of NE in patients with AR and/or asthma. A funnel plot was created to evaluate publication bias. A symmetrical appearance indicated low publication bias (Begg's test, P = 0.73; Egger test, P = 0.75) (Fig. 6A). Three RCTs [28,29,34] compared NEs between patients taking budesonide and those taking montelukast. No heterogeneity was observed (I^2^ < 50 %); therefore, a fixed effects model was used. This result revealed no statistically significant difference (RR 4.0 [95 % CI 0.47–34.20]; P = 0.817) (Fig. 6B). The remaining two RCTs [25,26] with 785 patients (experimental, n = 392; control, n = 393) were used to compare the incidence of NEs between patients taking loratadine and those taking montelukast. The heterogeneity test results were I^2^ = 60.7 %, P > 0.1. A random-effect model was used to combine the effect, with an RR of 0.50 (95 % CI 0.24–1.06; P = 0.111) (Fig. 6C). Furthermore, montelukast combined with loratadine likely caused more NEs than loratadine alone.

## 4 Discussion

Asthma and AR are common allergic diseases with high occurrence rates. Clinically, montelukast is used to treat asthma and AR [35,36]. Montelukast was approved by the FDA in 1998 for the treatment of asthma and, in 2002, for the treatment of AR; however, long-term use of montelukast can cause adverse neuropsychiatric reactions [35]. In 2009, the FDA added NEs to treat ADRs. With the increase in reports of NEs, the FDA strengthened existing warnings about serious behavior- and mood-related changes in montelukast on March 4, 2020 [[15], [16], [17]].

However, only pharmacovigilance studies [[4], [5], [6],^8^,[11], [12], [13], [14]] and case reports [7,9,10] have shown that montelukast can cause adverse neuropsychiatric reactions. High-quality randomized controlled studies have not been comprehensively analyzed. Therefore, this meta-analysis aimed to compare whether montelukast causes NEs in patients with asthma, rhinitis, or both. Eleven RCTs [1,[21], [22], [23], [24], [25], [26], [27],^30^,^31^,^33^] comprising 4402 patients were included in our meta-analysis. The results indicated no statistically significant differences between montelukast and NEs in either asthma or AR. In addition, the most common neuropsychiatric adverse reaction―namely, headache―was also analyzed. The analysis revealed that montelukast did not significantly increase the incidence of headaches. These results were consistent with the overall results of the included studies.

Aldea-Perona et al. [5] found that age differences in the occurrence of NEs and neuropsychiatric diseases are more common in children than in adults. Therefore, we grouped patients according to age to analyze its role in the occurrence of NEs. The relationship between montelukast and NEs in children (≤14 years of age) and adults (≥15 years of age) was analyzed using the medical concept of adult age. The results yielded an RR of 0.87 (95 % CI of 0.73–1.02; P = 0.537). This indicated that there was no significant difference between children and adults, and no obvious correlation between montelukast and NEs in children. This is consistent with the results of a large, nested, case-control study with 1920 patients with asthma matched for age, sex, and geographical region [8], which demonstrated no significant positive association between montelukast use and NEs among children. However, the results differed from those of other reports and databases. Approximately 23 % of the reported ADRs to montelukast occur in patients <11 years of age on the World Health Organization's (WHO) VigiAccess database (https://www.vigiaccess.org/). NE events mentioned in the WHO's VigiAccess database include nervous system disorders (headache, dizziness, somnolence, tremor, etc.) and psychiatric disorders (depression, anxiety, insomnia, suicidal ideation, etc.), with headache being the most common nervous system disorder. The FDA has boxed warnings about serious mental health side effects (risks may include suicidal thoughts or actions) for asthma and the allergy drug montelukast, mainly based on case reports submitted to the FDA. In this study, we screened all NEs associated with montelukast and found that headache was the most common NE in all RCT studies, although our analysis did not reveal a relationship between NE and montelukast. In addition, the high incidence of such NEs among adults and children in the United States, Canada, and Europe [37] suggests that montelukast may lead to NEs. Consequently, montelukast-associated NEs have drawn much attention from practicing pediatricians in the United States, Australia, and the United Kingdom. The risk for depression associated with montelukast use has been reported in the United States [38] and Turkish [39] guidelines. Dixon et al. reported that montelukast causes ADRs, such as neuropsychiatric and gastrointestinal disorders, in children and young individuals with asthma; however, this systematic review was mainly based on case reports, case-control studies, and cohort studies (only 1 RCT) [40]. It should be noted that most reported NE cases were from retrospective studies; furthermore, the results of this study based on RCTs could not fully cover NEs because there were too few RCTs addressing ADRs in children. Nevertheless, assessment before medication and monitoring during drug administration are essential in some special and sensitive populations [41]. Given the inconsistency between our results, based on RCTs, and the current large number of case reports, we further grouped the follow-up time and found a correlation between NEs and montelukast in the group with a follow-up > 1 month, with a small bias (Bisgaard, 2009). We speculate that with longer follow-up periods and more RCTs, the relationship between NEs and montelukast may become clearer. This also heightens the importance of explaining the potential adverse effects to parents before prescribing montelukast to their children. Risk management plans should be carefully explained. Pediatric healthcare providers should be aware of the risk for NEs associated with montelukast use and advise patients to observe, monitor, and report possible NEs.

We also analyzed the occurrence of adverse events associated with montelukast combined with commonly used drugs for AR or asthma, such as loratadine and budesonide. Loratadine is an H1 receptor antagonist that is used to treat rhinitis and asthma. Our analysis demonstrated that loratadine combined with montelukast significantly relieved rhinitis and asthma [42]. When combined with montelukast or double doses, the hormone medication budesonide demonstrated similar improvement among patients [28]. To clarify the relationship between montelukast and the occurrence of adverse mental events, we analyzed AR and asthma. Taking loratadine or inhalation of budesonide and montelukast in combination or alone, the occurrence of NEs was not statistically significant (P > 0.05) in the montelukast group compared with the control group. Combining medications may be more effective, considering the effects of medication and the side effects of hormone drugs.

Our results revealed no significant association between montelukast use and NEs in patients with rhinitis or asthma. We hypothesized that NE is associated with this disease. Timonen et al. [43] and Kovacs et al. [44] found that individuals with allergies were more likely to develop depression than healthy individuals. This finding may be due to disorders of the hypothalamic-pituitary-adrenal axis and sympathetic adrenal medulla systems, and changes in the secretion of cytokines in the body, which cause patients to be affected by emotional and behavioral factors in daily life, depression, and other neuropsychiatric adverse events. Asthma is a chronic disease and repeated attacks can result in severe mental disorders. The inflammatory state of the body during the onset of asthma leads to an increase in proinflammatory cytokines, which are associated with the development of depression [45]. In addition, animal studies have shown that after increasing the cholinergic response in rats, tracheal constriction and airway inflammation may develop, thereby increasing the occurrence of NEs [46,47]. The above-mentioned research indicates that NEs may be caused by airway allergic diseases. However, we were unable to conduct further research owing to insufficient data.

All except 1 of the studies [32] were of good quality according to Begg and Egger tests. The major funnel plots were symmetrical, indicating no evidence of publication bias. Our meta-analysis revealed no significant increase in the incidence of NEs in patients with AR and asthma compared with the placebo group (P > 0.05).

Findings of the present study have practical implications for physicians, patients (especially parents of child patients), and regulatory agencies. As a leukotriene receptor antagonist, montelukast has been warned by the FDA; however, it is still of great value in the treatment of AR and asthma, and there is currently no ideal alternative medication. It must be emphasized that this drug should be used with strict indications and for an appropriate duration. At the same time, adverse effects of NE must be strictly monitored during montelukast use.

## 5 Strengths and limitations

A major strength of our study was that it was the first RCT-based systematic review of the correlation between montelukast and NEs. It has been established that RCTs represent the highest level of original research because they use random assignment methods that control for confounding factors and provide strong evidence supporting cause and effect.

We must, however, acknowledge that the present study also had some limitations. First, the effect of disease on the results should not be excluded. Second, because the study was not designed to report ADRs, no detailed records of their occurrence were available. Third, there are too few RCTs addressing ADRs in children to fully cover all NEs. Finally, observational studies were excluded from the analyses. Data from some pharmacovigilance research databases [6,7,[11], [12], [13], [14], [15],^17^] could not be combined and analyzed, which further reduced the reliability of the studies’ results and affected study quality.

## 6 Conclusions

In conclusion, the present study found no cause-and-effect relationship between montelukast and NEs in patients with asthma and AR based on current evidence from RCTs. However, there is an increased incidence of NEs when montelukast was combined with loratadine, or when the follow-up was >1 month (with a little bias). To our knowledge, this is the first meta-analysis-based RCT to provide evidence of this aspect, and our results are not consistent with those of some current cohort-based studies. Whether this is because RCT studies, as high-level evidence-based medical research, remain few in number. Anyway, it definitely does not mean we disagree with the reports of others, it only indicates that the issue should be taken more seriously. Therefore, large-sample RCTs are needed to verify the association between NEs and montelukast use in patients with allergic airway disease, and attention should also be devoted to FDA warnings.

## Funding

This work was supported by the 10.13039/501100001809National Natural Science Foundation of China (82071021) and Shandong Provincial Postdoctoral Foundation Project (Innovation Project) (SDCX-ZG-202203077).

## Ethics approval and consent to participate

Informed consent was not needed for this study because all analyses were based on previous published studies.

## Consent for publication

Not applicable.

## Data availability statement

Data included in article/supp. Material/referenced in article.

## CRediT authorship contribution statement

**Yakui Mou:** Writing – review & editing, Writing – original draft, Data curation, Conceptualization. **Qing Song:** Writing – original draft, Data curation. **Chunying Zhao:** Writing – original draft, Data curation. **Han Fang:** Methodology, Formal analysis. **Chao Ren:** Methodology, Formal analysis, Conceptualization. **Xicheng Song:** Writing – review & editing, Investigation, Funding acquisition, Conceptualization.

## Declaration of competing interest

The authors declare that they have no known competing financial interests or personal relationships that could have appeared to influence the work reported in this paper.

## References

[1] Baena-Cagnani C.E., Berger W.E., DuBuske L.M. (2003). Comparative effects of desloratadine versus montelukast on asthma symptoms and use of beta 2-agonists in patients with seasonal allergic rhinitis and asthma. Int. Arch. Allergy Immunol..

[2] Price D.B., Hernandez D., Magyar P. (2003). Randomised controlled trial of montelukast plus inhaled budesonide versus double dose inhaled budesonide in adult patients with asthma. Thorax.

[3] Reiss T.F., Chervinsky P., Dockhorn R.J., Shingo S., Seidenberg B., Edwards T.B. (1998). Montelukast, a once-daily leukotriene receptor antagonist, in the treatment of chronic asthma: a multicenter, randomized, double-blind trial. Montelukast Clinical Research Study Group. Arch. Intern. Med..

[4] Aagaard L., Hansen E.H. (2014). Paediatric adverse drug reactions following use of asthma medications in Europe from 2007 to 2011. Int. J. Clin. Pharm..

[5] Aldea Perona A., Garcia-Saiz M., Sanz Alvarez E. (2016). Psychiatric disorders and montelukast in children: a disproportionality analysis of the VigiBase((R)). Drug Saf..

[6] Bygdell M., Brunlof G., Wallerstedt S.M., Kindblom J.M. (2012). Psychiatric adverse drug reactions reported during a 10-year period in the Swedish pediatric population. Pharmacoepidemiol. Drug Saf..

[7] Callero-Viera A., Infante S., Fuentes-Aparicio V., Zapatero L., Alonso-Lebrero E. (2012). Neuropsychiatric reactions to montelukast. J Investig. Allergol. Clin. Immunol..

[8] Cereza G., Garcia Dolade N., Laporte J.R. (2012). Nightmares induced by montelukast in children and adults. Eur. Respir. J..

[9] Ibarra-Barrueta O., Palacios-Zabalza I., Mora-Atorrasagasti O., Mayo-Suarez J. (2014). Effect of concomitant use of montelukast and efavirenz on neuropsychiatric adverse events. Ann. Pharmacother..

[10] Kocyigit A., Gulcan Oksuz B., Yarar F., Uzun F., Igde M., Islek I. (2013). Hallucination development with montelukast in a child with asthma: case presentation. Iran. J. Allergy, Asthma Immunol..

[11] Lafay-Chebassier C., Chavant F., Favreliere S., Pizzoglio V., Perault-Pochat M.C. (2015). Drug-induced depression: a case/non case study in the French pharmacovigilance database. Therapie.

[12] B P., W L., P G., F S., S S., Mann R.D. (2001). Pharmacosurveillance and safety of the leukotriene receptor antagonist (LTRA), montelukast. Clin. Exp. Allergy Rev..

[13] Marchand M.S., Jonville-Bera A.P., Autret-Leca E. (2013). [Psychiatric disorders associated with montelukast: data from the National Pharmacovigilance Database]. Arch. Pediatr. : organe officiel de la Societe francaise de pediatrie.

[14] Wallerstedt S.M., Brunlof G., Sundstrom A., Eriksson A.L. (2009). Montelukast and psychiatric disorders in children. Pharmacoepidemiol. Drug Saf..

[15] US Food and Drug Administration (2020). https://www.fda.gov/drugs.

[16] US Food and Drug Administration (2008). http://www.fda.gov/Drugs.

[17] US Food and Drug Administration (2009). http://www.fda.gov/Drugs.

[18] Narang R., Narang S., Narang D., Udeani G. (2014). Contemporary use of montelukast and its association with depression in asthma and allergic rhinitis patients. Chest.

[19] Jadad A.R. (1998).

[20] Higgins J., Green S. (2011).

[21] Knorr B., Matz J., Bernstein J.A. (1998). Montelukast for chronic asthma in 6- to 14-year-old children: a randomized, double-blind trial. Pediatric Montelukast Study Group. JAMA.

[22] Leff J.A., Busse W.W., Pearlman D. (1998). Montelukast, a leukotriene-receptor antagonist, for the treatment of mild asthma and exercise-induced bronchoconstriction. N. Engl. J. Med..

[23] Noonan M.J., Chervinsky P., Brandon M. (1998). Montelukast, a potent leukotriene receptor antagonist, causes dose-related improvements in chronic asthma. Montelukast Asthma Study Group. Eur. Respir. J..

[24] Malmstrom K., Rodriguez-Gomez G., Guerra J. (1999). Oral montelukast, inhaled beclomethasone, and placebo for chronic asthma. A randomized, controlled trial. Montelukast/Beclomethasone Study Group. Ann. Intern. Med..

[25] Meltzer E.O., Malmstrom K., Lu S. (2000). Concomitant montelukast and loratadine as treatment for seasonal allergic rhinitis: a randomized, placebo-controlled clinical trial. J. Allergy Clin. Immunol..

[26] Nayak A.S., Philip G., Lu S., Malice M.P., Reiss T.F. (2002). Efficacy and tolerability of montelukast alone or in combination with lo-ratadine in seasonal allergic rhinitis: a multicenter, randomized, double-blind, placebo-controlled trial performed in the fall. Ann. Allergy Asthma Immunol.: official publication of the American College of Allergy, Asthma, & Immunology.

[27] Baumgartner R.A., Martinez G., Edelman J.M. (2003). Distribution of therapeutic response in asthma control between oral montelukast and inhaled beclomethasone. Eur. Respir. J..

[28] Yildirim Z., Ozlu T., Bulbul Y., Bayram H. (2004). Addition of montelukast versus double dose of inhaled budesonide in moderate persistent asthma. Respirology.

[29] Shah A.R., Sharples L.D., Solanki R.N., Shah K.V. (2006). Double-blind, randomised, controlled trial assessing controller medications in asthma. Respiration; international review of thoracic diseases.

[30] (2007). Clinical trial of low-dose theophylline and montelukast in patients with poorly controlled asthma. Am. J. Respir. Crit. Care Med..

[31] Bisgaard H., Skoner D., Boza M.L. (2009). Safety and tolerability of montelukast in placebo-controlled pediatric studies and their open-label extensions. Pediatr. Pulmonol..

[32] Prenner B.M., Lu S., Danzig M.R. (2010). Safety of fixed-dose loratadine/montelukast in subjects with allergic rhinitis. Allergy Asthma Proc..

[33] Reiss T.F., Chervinsky P., Dockhorn R.J. (1998). Montelukast, a once-daily leukotriene receptor antagonist, in the treatment of chronic asthma: a multicenter, randomized, double-blind trial. Montelukast Clinical Research Study Group. Arch. Intern. Med..

[34] Chen H., Zhang L., Lou H.F. (2021). A randomized trial of comparing a combination of montelukast and budesonide with budesonide in allergic rhinitis. The laryngoscope.

[35] Bernstein J.A. (2010). Allergic and mixed rhinitis: epidemiology and natural history. Allergy Asthma Proc..

[36] Martinez F.D., Vercelli D. (2013). Asthma. Lancet (London, England).

[37] Al-Shamrani A., Alharbi S., Kobeisy S. (2022 Nov 21). Adverse drug reactions (ADRs) of montelukast in children. Children.

[38] Seidman M.D., Gurgel R.K., Lin S.Y. (2015). AAO-HNSF. Clinical practice guideline: allergic rhinitis. Otolaryngol. Head Neck Surg..

[39] Ecevit M.C., Özcan M., Haberal Can I. (2021). Turkish guideline for diagnosis and treatment of allergic rhinitis (ART). Turk. Arch. Otolaryngol..

[40] Dixon E.G., Rugg-Gunn C.E., Sellick V. (2021 Oct 13). Adverse drug reactions of leukotriene receptor antagonists in children with asthma: a systematic review. BMJ Paediatr Open.

[41] Mou Y.K., Wang H.R., Zhang W.B. (2022 Jan 4). Allergic rhinitis and depression: profile and proposal. Front. Psychiatr..

[42] Dockhorn R.J., Bergner A., Connell J.T. (1987). Safety and efficacy of loratadine (Sch-29851): a new non-sedating antihistamine in seasonal allergic rhinitis. Ann. Allergy.

[43] Timonen M., Jokelainen J., Silvennoinen-Kassinen S. (2002). Association between skin test diagnosed atopy and professionally diagnosed depression: a Northern Finland 1966 Birth Cohort study. Biol. Psychiatr..

[44] Kovacs M., Stauder A., Szedmak S. (2003). Severity of allergic complaints: the importance of depressed mood. J. Psychosom. Res..

[45] Eyre H., Baune B.T. (2012). Neuroplastic changes in depression: a role for the immune system. Psychoneuroendocrinology.

[46] Djuric V.J., Cox G., Overstreet D.H., Smith L., Dragomir A., Steiner M. (1998). Genetically transmitted cholinergic hyperresponsiveness predisposes to experimental asthma. Brain Behav. Immun..

[47] Overstreet D.H., Daws L.C., Schiller G.D., Orbach J., Janowsky D.S. (1998). Cholinergic/serotonergic interactions in hypothermia: im-plications for rat models of depression. Pharmacol., Biochem. Behav..

