# Supplementary material for: Meta-analysis of the relationship between montelukast use and neuropsychiatric events in patients with allergic airway disease
Source: Heliyon. 2023 Nov 8;9(11):e21842. doi: 10.1016/j.heliyon.2023.e21842 (PMC10685197; doi:10.1016/j.heliyon.2023.e21842)
Supplement: Multimedia component 1 [file mmc1.docx]

**Outcome measures**

We used the WHO 's VigiAccess database (https://www.vigiaccess.org/) about the definition of neuropsychiatric as the index of primary outcome measurement. It includes nervous system disorders (headache; dizziness; somnolence; tremor; paraesthesia; disturbance in attention; psychomotor hyperactivity; seizure; hypoaesthesia; memory impairment; migraine; amnesia; lethargy; speech disorder; loss of consciousness; syncope; neuropathy peripheral; dysgeusia; dyskinesia; balance disorder; burning sensation; sedation; epilepsy; generalized tonic-clonic seizure; cognitive disorder; hypersomnia; mental impairment; nervous system disorder; aphasia; cerebrovascular accident; movement disorder; sensory disturbance; formication; depressed level of consciousness; dysarthria; hypotonia; neuralgia; presyncope; hyperkinesia; restless legs syndrome; etc) and psychiatric disorders(depression; anxiety; insomnia; suicidal ideation; aggression; nightmare; abnormal behavior; irritability; anger; agitation; sleep disorder; hallucination; mood swings; mood altered; depressed mood; suicide attempt; abnormal dreams ; neuropsychiatric symptoms; sleep terror; restlessness; fear; emotional disorder; tic; panic attack; nervousness; mental disorder; intentional self-injury; confusional state; personality change; obsessive-compulsive disorder; completed suicide; thinking abnormal; somnambulism; hallucination, visual; attention deficit hyperactivity disorder; middle insomnia; social avoidant behavior; poor quality sleep ; paranoia; enuresis; affective disorder; hallucination, auditory; disorientation; behavior disorder; self-injurious ideation; etc) two aspects.
